# A Mechanism of Action Study on Danggui Sini Decoction to Discover Its Therapeutic Effect on Gastric Cancer

**DOI:** 10.3389/fphar.2020.592903

**Published:** 2021-01-11

**Authors:** Boyu Pan, Yun Wang, Chunnuan Wu, Junrong Jia, Chen Huang, Senbiao Fang, Liren Liu

**Affiliations:** ^1^ Department of Gastrointestinal Cancer Biology, National Clinical Research Center for Cancer, Key Laboratory of Cancer Prevention and Therapy, Tianjin’s Clinical Research Center for Cancer, Tianjin Medical University Cancer Institute and Hospital, Tianjin, China; ^2^ Department of Integrated Traditional and Western Medicine, National Clinical Research Center for Cancer, Key Laboratory of Cancer Prevention and Therapy, Tianjin’s Clinical Research Center for Cancer, Tianjin Medical University Cancer Institute and Hospital, Tianjin, China; ^3^ Department of Pharmacy, National Clinical Research Center for Cancer, Key Laboratory of Cancer Prevention and Therapy, Tianjin’s Clinical Research Center for Cancer, Tianjin Medical University Cancer Institute and Hospital, Tianjin, China; ^4^ Public Laboratory, National Clinical Research Center for Cancer, Key Laboratory of Cancer Prevention and Therapy, Tianjin’s Clinical Research Center for Cancer, Tianjin Medical University Cancer Institute and Hospital, Tianjin, China; ^5^ School of Information Science and Engineering, Central South University, Changsha, China

**Keywords:** Danggui Sini Decoction, Chinese herb medicine, network pharmacology, gastric cancer, Akt/ERK/p53 signaling pathways, MCM2, new use of old drugs

## Abstract

Danggui Sini Decoction (DSD), a classic Chinese herb medicine (CHM) formula, has been used to treat various diseases in China for centuries. However, it remains challenging to reveal its mechanism of action through conventional pharmacological methods. Here, we first explored the mechanism of action of DSD with the assistance of network pharmacology and bioinformatic analysis tools, and found a potential therapeutic effect of DSD on cancer. Indeed, our *in vivo* experiment demonstrated that oral administration of DSD could significantly inhibit the growth of xenografted gastric cancer (GC) on mice. The subsequent enrichment analyses for 123 candidate core targets evacuated from the drug/disease-target protein-protein interaction network showed that DSD could affect the key biological processes involving the survival and growth of GC cells, such as apoptosis and cell cycle, and the disturbance of these biological processes is likely attributed to the simultaneous inhibition of multiple signaling pathways, including PI3K/Akt, MAPK, and p53 pathways. Notably, these *in silico* results were further validated by a series of cellular functional and molecular biological assays *in vitro*. Moreover, molecular docking analysis suggested an important role of MCM2 in delivering the pharmacological activity of DSD against GC. Together, these results indicate that our network pharmacology and bioinformatics-guided approach is feasible and useful in exploring not only the mechanism of action, but also the “new use” of the old CHM formula.

## Introduction

Characterized by its holistic concept and the theory of Zheng (syndrome) differentiation, Traditional Chinese medicine (TCM) has been widely used to treat various diseases in China for thousands of years (Tang et al., 2008). As a key component of TCM, Chinese herbal medicine (CHM) offers a promising perspective for treating complex diseases such as cancer owing to its “multi-ingredient, multi-target, and multi-function” pharmacological characteristics (Ling et al., 2014; Liu et al., 2016). To elucidate the action mechanism of CHM with a better understanding of how the multiple ingredients in CHM act in synergy, and what effects they exert on multiple targets, may shed some light on the discovery of “new uses of old CHM,” and accelerate the transformation of TCM from an experience-based medicine system into an evidence-based medicine system. However, to reveal the scientific basis of CHM at the molecular level and in a systematic manner is still a great challenge when using conventional approaches, as the diversity of the ingredients in CHM and the complexity of the diseases themselves greatly complicate the mechanism of action study on CHM (Hsiao and Liu, 2010; Li and Zhang, 2013).

Recently, with advances in systems biology and bioinformatics, network pharmacology has become more prominent and significantly reshaped the paradigm of drug mechanism studies (Hopkins, 2007; Hopkins, 2008). Derived from network pharmacology, TCM network pharmacology was recently launched along with the introduction of a “network target” concept, which considers the disease network composed of numerous biomolecules as a whole target. In light of this, it may provide not only a deeper insight into the action mechanism of CHM in a holistic view, but also a potential tool to explore new applications of CHM in a systematic manner (Li, 2011; Li and Zhang, 2013).

**FIGURE 1:**
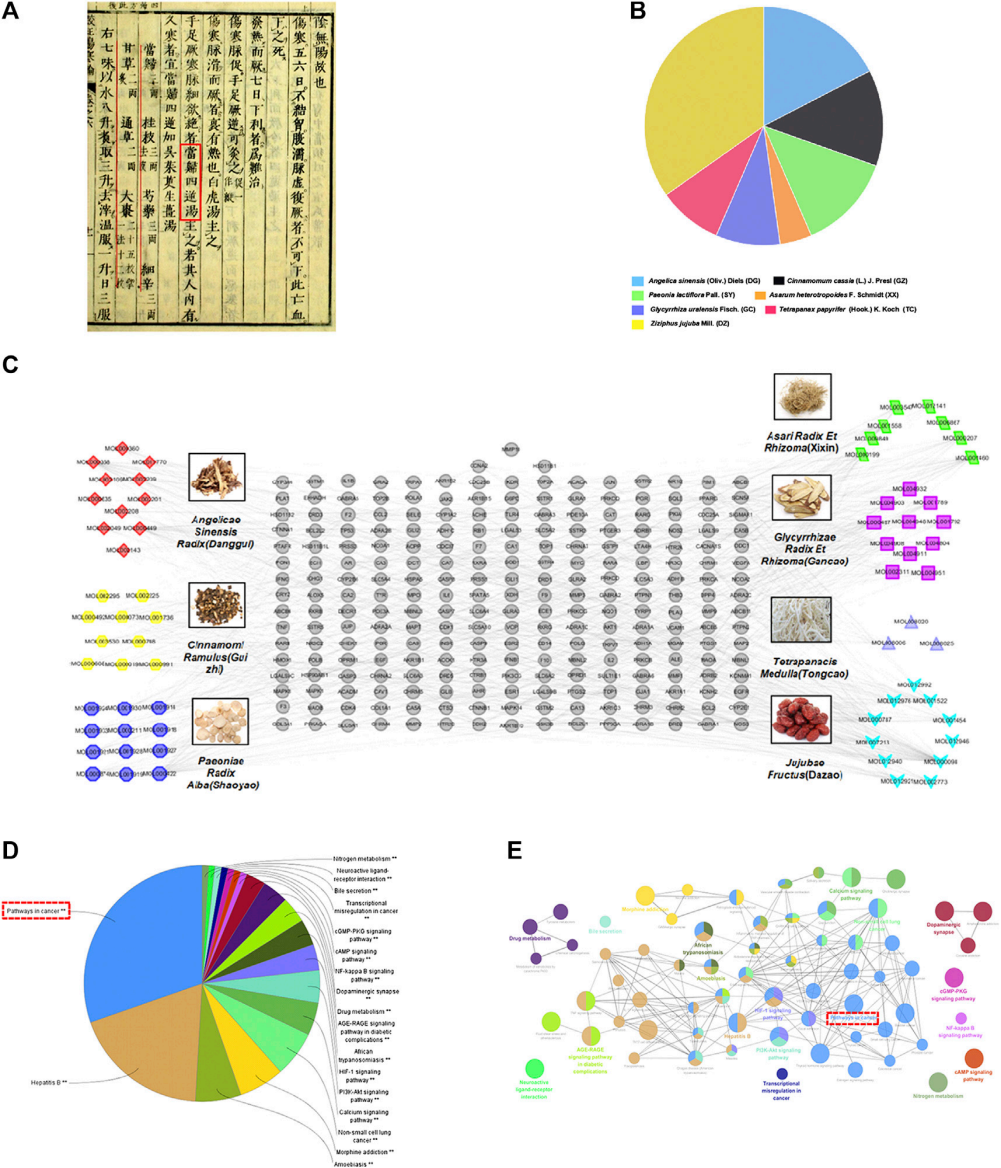
Construction of the DSD candidate active ingredient-putative target network and enrichment analysis. **(A)** Danggui Sini Decoction (DSD) obtained from the Treatise on Febrile Diseases (Han Dynasty in ancient China). **(B)** The quality matching of seven important pharmaceutical ingredients from DSD (DG, GZ, SY, XX, GC, TC, and DZ). **(C)** This complex and systematic network was constructed by linking the candidate active ingredients and their putative targets of the seven herbs, which were constituents of DSD. **(D,E)** Putativedrug targets were enriched in the representative signaling pathways using ClueGO plugin (*p* < 0.05).

Danggui Sini Decoction (DSD), a classic CHM formula from Treatise on Febrile Diseases (Figure 1A), was recently promulgated as one of the top 100 Classical CHM Prescriptions in China. DSD is composed of seven herbs, namely *Angelica sinensis* (Oliv.) Diels (Danggui, DG), *Cinnamomum cassia* (L.) J. Presl (Guizhi, GZ), Paeonia lactiflora Pall. (Shaoyao, SY), Asarum heterotropoides F. Schmidt (Xixin, XX), *Glycyrrhiza* uralensis Fisch. (Gancao, GC), Tetrapanax papyrifer (Hook.) K. Koch (Tongcao, TC), and Ziziphus jujuba Mill. (Dazao, DZ) (Figure 1B). The above seven species were fully validated using “The Plant List” key search tool (www.theplantlist.org). According to TCM theory, DSD is traditionally used to treat TCM syndromes such as blood-deficiency and cold-coagulation-meridian, due to its functions in activating blood flow and dissipating blood stasis and chills. Recent studies showed that it exhibited desirable pharmacological effects on hypoxic injury of islet endothelial cells and peripheral neuropathy caused by chronic chemotherapy (Liu et al., 2017; Chen et al., 2018). However, the action mechanism of DSD, as well as its potential applications on other diseases, has yet to be systematically investigated.

In this study, taking advantage of the TCM network pharmacology database, we first performed an *in silico* study to explore the action mechanism of DSD, which suggested a novel application of DSD on cancer treatment. The subsequent bioinformatics-guided *in vitro* and *in vivo* assays demonstrated a direct inhibitory effect of DSD on the growth of gastric cancer (GC) cells via simultaneously impeding multiple signaling pathways. Thus, our results provide an insight into exploring novel clinical applications for CHM formula via network pharmacology and a bioinformatics-guided approach.

## Materials and Methods

### Cell Culture and Reagents

Human GC SGC-7901 and AGS cells were obtained from the National Infrastructure of Cell Line Resources (Beijing, China) and maintained in RPMI-1640 and F12K medium, respectively, and supplemented with 10% (v/v) FBS and 100 U/ml streptomycin/penicillin in a humidified atmosphere of 5% CO_2_ at 37°C. RPMI-1640 medium and F12K medium were purchased from GIBCO (United States). Fetal bovine serum (FBS) was purchased from ThermoFisher (United States). Cell Counting Kit-8 (CCK-8) reagent was bought from Dojindo (Japan). Hoechst 33342 reagent was obtained from Beyotime (China). Annexin-V FITC apoptosis detection kit (#556547) and FITC BrdU Flow kit-PartA (#559619) were purchased from BD Biosciences Pharmingen (United States). Antibodies against Bcl-2, Bax, Caspase 9/p35/p10, Cyclin D1, Cyclin A2, and beta-actin were bought from Proteintech. Antibodies against Cyclin B1, p-Akt (T308), p-ERK (T202/Y204), and p-p53 (S392) were bought from CST. Antibodies against CDK2 was purchased from Abcam. The above seven herbs were collected from Tianjin Medical University Cancer Institute and Hospital TCM Pharmacy, and they were stored in a dry environment away from light. Meanwhile, the above herbs were also kept for posterity, and they can be accessed from our TCM Pharmacy in the future. Meanwhile, DSD was also provided by our TCM Pharmacy, and was prepared in line with the China Pharmacopoeia standard of quality control. The quality matching (g) of these seven important pharmaceutical ingredients from DSD was as follows: DG: GZ: SY: XX: GC: TC: DZ = 4: 3: 3: 1: 2: 2: 8. The final concentration of DSD was 0.03 g/ml. For biological studies, the 0.03 g/ml nominal concentration decoction was filtered (0.22 µm), sterilized, and diluted. Furthermore, the 0.03 g/ml liquor was also normally stored in an ultra-low temperature environment (−80 °C) for posterity.

### Candidate Ingredients Composition of Danggui Sini Decoction

The chemical composition of all seven herbs that constitute DSD was mainly obtained from Traditional Chinese Medicine Systems Pharmacology (TCMSP) Database (http://lsp.nwu.edu.cn/tcmsp.php), and the parameters for selection of the active ingredients were set as oral bioavailability (OB) ≥30% and drug-likeness (DL) ≥0.18 as standard (Pei et al., 2016; Zhang et al., 2016). Furthermore, we used the literature-mining method (www.CNKI.net) to search for those that had been reported as the key ingredients of the herbs, although they failed to meet the above parameters.

### Identification of Putative Drug Targets and Known Gastric Cancer-Related Targets

The systematic drug targeting approach was utilized to identify potential targets for the medicinal composition of DSD (Yu et al., 2012). The potential drug targets were obtained from TCMSP and Swiss Target Prediction databases (http://www.swisstargetprediction.ch/) (see Supplementary Table S1 for details). The known GC-related targets were obtained from Gene Expression Omnibus (GEO) database (see Supplementary Table S2 for details). Three gene expression datasets (GSE33335, GSE54129, and GSE79973), derived from human GC and adjacent normal tissue, were included. The protein-protein interaction (PPI) data were analyzed using Bisogenet, a plugin of Cytoscape, and the final result was integrated into a single graph from six analyses of the obtained PPI datasets.

### Network Construction and Enrichment Analysis

The interaction networks for putative drug targets of DSD and the known GC-related targets based on the data obtained from the Cytoscape plugin Bisogenet were constructed and visualized using Cytoscape (Version 3.2.1) (Shannon et al., 2003). After merging of the above two networks, the topology parameter of each node in the merged network was calculated using CytoNCA, another plugin of Cytoscape. The node with a score two times higher than the median of “Degree centrality” (DC) was considered important and appeared in the new network. Next, to further screen out the core targets, the node with a score higher than the median of DC, “Betweenness centrality” (BC), and “Closeness centrality” (CC) were considered significant. The definitions and computational formulas of the above parameters have been previously defined (Pan et al., 2018a) (see Supplementary Table S3 for details).

Functional and pathway enrichment analyses of the obtained putative and core targets were performed using ClueGO (a key plugin in Cytoscape), The Database for Annotation, Visualization and Integrated Discovery (DAVID) v6.8 (https://david.ncifcrf.gov/), and OmicShare online tools (http://www.omicshare.com/tools) (Huang et al., 2009a; Huang et al., 2009b). The analyses were divided into two types: GO biological process and KEGG signaling pathways.

### Xenograft Mouse Work

Four week old BALB/c nude mice were purchased from Beijing Vital River Laboratory Animal Technology Co., Ltd. (China). All procedures for the animal experiments were conducted according to the Animal Ethics Committee of Tianjin Medical University Cancer Institute and Hospital. At 5 weeks of age, SGC-7901 cells (2 × 10^7^/ml) were inoculated subcutaneously into the right flanks of 24 mice using 1 ml needles. When the tumors were visible by the naked eye at approximately 10 days after the inoculation, the 24 mice were randomly divided into four groups of six mice each. DSD was orally gavaged with a single dose of 200 µl (300 mg/kg) for 14 consecutive days twice per day. In the control group, mice were orally administered with the same volume of saline twice per day. Also, low doses of oxaliplatin (L-OHP, 4 mg/kg) plus 5-fluorouracil (5-FU, 20 mg/kg) and high doses of L-OHP (8 mg/kg) plus 5-FU (20 mg/kg) were administered intraperitoneally once every three days to two other groups, respectively (both L-OHP and 5-FU were diluted with 5% glucose). During the experiments, the tumor volumes were measured daily using the following formula: long diameter × (short diameter) 2/2. On day 14 of the intervention, mice were sacrificed and the tumor tissues were weighed. None of the mice died during the experiments.

### Immunohistochemistry Assays

The slides of tumor tissue sections were disposed of with deparaffinization and antigen unmasking, and were then incubated with the antibody against Ki-67 (Abcam, United Kingdom) at 4 C overnight. After washing with PBS, the slides were incubated with Polymer Helper and Poly peroxidase-anti-mouse/rabbit IgG (PV-9000, ORIGENE, China), followed by further incubation with diaminobenzidine (DAB). The tissue sections were mounted after being counterstained with hematoxylin. Some sections were stained with H&E for the histological analysis.

### Cellular Functional Assays

The cell viability was assessed by the CCK-8 assay, in which 10 μl of CCK-8 solution was added to each well, and the cells were incubated for an additional 2 h before the absorbance value was measured at a wavelength of 450 nm using an automated microplate reader. The xCELLigence RTCA instrument was used to further evaluate drug toxicity. Measurements were taken continuously for ∼48 h at 37°C, and the RTCA software was used for subsequent data analysis. Cell colony formation assay was carried out for 14 days and cell colonies were counted after being stained with 0.1% crystal violet. The accumulated distance of cells were acquired on the Operetta CLS High Content Analysis System equipped with Harmony software (PerkinElmer, Waltham, MA, United States) using a ×20 long wide distance objective in a digital phase contrast mode at a temperature of 37°C and 5% CO_2_. Cell motility was monitored by time-lapse image sequencing for 24 h. Cell morphology was observed in white light and fluorescence (staining cell nucleus with Hoechst 33342 reagent) field using an inverted microscope. Apoptosis detection and cell cycle assay were performed according to the manufacturer’s instructions. The details are as follows: First, the cells were seeded at a density of 10^5^ cells/well in 6-well plates after being treated with different concentrations of DSD. Next, the cells were subsequently stained using an Annexin-V FITC apoptosis detection kit and FITC BrdU Flow kit-PartA. Staining was performed according to the manufacturer’s protocol. The cells were then determined using a FACSCalibur flow cytometer (BD Biosciences, Franklin Lakes, NJ, United States).

### Liquid Chromatography, Mass Spectrometric Conditions, and Sample Extraction Preparation

Liquid chromatographic separation and mass spectrometric detection were performed using Waters Quattro Premier XE/Acquity UPLC system coupled to a tandem quadrupole mass spectrometer (Waters Corporation, Milford, MA, United States). Lab solutions LCMS software (Masslynx V4.1) was used to control the instruments and process the data. This instrument was equipped with both ESI and APCI sources. Please see more details as we previously described (Pan et al., 2019).

### Molecular Docking Analysis

AutoDock software (4.2 versions) was used to dock the structures of seven ingredients, including senkyunolide A, senkyunolide G, senkyunolide I, ferulic acid, ferulaldehyde, O-Methoxycinnamal, and cinnamic acid from monarch drug (DG and GZ) in DSD detected by LC-MS. These ingredients were docked with EGFR (PDB code: 3W2S), CUL3 (PDB code: 5JA4), APP (PDB code: 5BUO), MCM2 (PDB code: 3JA8), CDK2 (PDB code: 3MY5), and FN1 (PDB code: 3M7P), respectively. Using Autodock, the protein targets and small molecules could be flexibly docked to obtain the initial docking conformation structure, and at least 40 docking conformation results would be generated. Next, among these 40 docking conformations, the one with the best phase of docking energy in each docking pair was selected for structure extraction and used for subsequent docking mode analysis. Finally, the energy values of 42 docking analyses between the seven ingredients and six hub targets were summed up.

### Statistical Analysis

All data were analyzed using SPSS17.0. software (United States). Results were represented as mean with standard deviations (mean ± SD). The differences were expressed using the Student’s *t*-test. A *p* < 0.05 was considered as statistically significant.

## Results

### *In silico* Active Ingredients and Drug Targets Screening for Danggui Sini Decoction

To explore the action mechanism of DSD, we first conducted a virtual study of combined OB screening with DL evaluation to identify the active ingredients in DSD. A total of 43 potential compounds with OB ≥ 30% and DL ≥ 0.18 from the herb constituents of DSD were obtained using TCMSP platform. Meanwhile, another 23 ingredients that failed to meet the above parameters (with OB < 30% or DL < 0.18), but either exhibited extensive pharmacological activities or were found to be typical ingredients of the herbs by literature mining, were also collected for subsequent analysis. These 66 compounds from the seven herbs in DSD were considered as the candidate ingredients. As shown in Table 1, the seven different herbs, namely DG, GZ, SY, XX, GC, TC, and DZ, contributed 11, 10, 12, 8, 11, 3, and 11 candidate ingredients, respectively.

**TABLE 1:** Active components identified in seven herbs of DSD.

Herbs	Number	Components
*Angelicae sinensis sadix* (Danggui, DG)	11	Beta-sitosterol, stigmasterol, senkyunolide A, senkyunolide G, senkyunolide I, senkyunolide J, ferulic acid, vanillin, Z-6,8′,7,3′-diligustilide, ferulaldehyde, Z-ligustilide
*Cinnamomi ramulus* (Guizhi, GZ)	10	(+)-catechin, ent-epicatechin, (−)-taxifolin, cinnamaldehyde, cinnamic alcohol, O-methoxycinnamaldehyde, cinnamic acid, D-camphene, ()-Terpinen-4-ol, benzaldehyde
*Paeoniae radix alba* (Shaoyao, SY)	12	Paeoniflorgenone, albiflorin_qt, paeoniflorin, lactiflorin, benzoyl paeoniflorin, kaempferol, mairin, (3S,5R,8R,9R,10S,14S)-3,17-dihydroxy-4,4,8,10,14-pentamethyl-2,3,5,6,7,9-hexahydro-1H-cyclopenta [a]phenanthrene-15,16-dione, paeonol, oxypaeoniflorin, gallotannin, albiflorin
*Asari radix Et rhizoma* (Xixin, XX)	8	Cryptopin, zinc05223929, sesamin, caribine, methyleugenol, α-pinene, safrol, asarone
*Glycyrrhizae radix Et rhizoma* (Gancao, GC)	11	Glabrene, glabridin, glycyrol, isoglycyrol, licochalcone a, liquiritigenin, liquiritin, 18beta-glycyrrhetinic acid, glycyrrhizin, isoliquiritigenin, isoliquiritin
*Tetrapanacis medulla* (Tongcao, TC)	3	Paryriogenin A, paryriogenin I, tetrapanoside B_qt
*Jujubae fructus* (Dazao, DZ)	11	Stepharine, spiradine A, quercetin, (S)-coclaurine, coumestrol, fumarine, berberine, mauritine D, beta-carotene, nuciferin, zizyphus saponin I_qt

Next, we explored the drug targets for the above candidate ingredients in DSD using TCMSP and SwissTargetPrediction databases, which resulted in a total of 243 putative targets for the 66 candidate ingredients (Figure 1C). The numbers of putative targets in DG, GZ, SY, XX, GC, TC, and DZ were 70, 68, 103, 71, 79, 17, and 129, respectively. Detailed information of these drug-related targets is listed in Supplementary Table S1. Notably, there were many overlapping targets among different ingredients, suggesting that they may play important roles in manifesting the synergistic effects of DSD. An individual systematic drug-target network was then constructed to visualize the complex interactions among these ingredients and their putative targets using Cytoscape 3.2.1 (see Supplementary Figure S1 for details).

### Signaling Pathway Enrichment Analysis Suggested a Novel Application of Danggui Sini Decoction on Cancer Treatment

To further clarify the action mechanism of DSD, we performed a KEGG signaling pathway enrichment analysis for these identified 243 putative drug targets by using ClueGO plugin (Figures 1D,E). Intriguingly, the analysis result showed that the most enriched signaling pathway was “pathways in cancer,” which for the first time connected DSD to cancer treatment. In addition, other signaling pathways were also enriched, such as Hepatitis B, Calcium signaling pathway, PI3K-Akt signaling pathway, and HIF-1 signaling pathway. Notably, some of the above signaling pathways have been previously reported to be involved in diseases that could be treated by DSD (You et al., 2009; Cheng et al., 2017; Chen et al., 2018). To confirm the novel role of DSD in cancer therapy, we further performed the disease enrichment analysis for the drug targets of DSD by GAD Disease Class using DAVID v6.8. The result showed that “cancer” was ranked the top fourth among the 16 enriched diseases (see Supplementary Figure S2 for details). Thus, these results not only helped to reveal the potential action mechanism of DSD, but also suggested a great potential of DSD in cancer treatment.

### Identification of the Related Targets for Gastric Cancer

**FIGURE 2:**
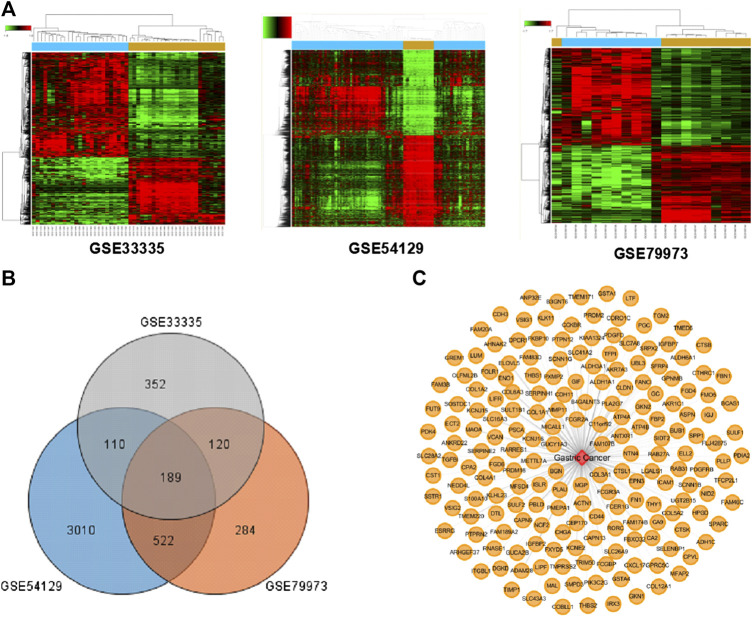
The known GC-related targets were obtained from the Gene Expression Omnibus (GEO) database. **(A)** Three heat maps from GEO chips, including GSE33335, GSE54129, and GSE79973. **(B)** The Venn diagram of 189 common GC-related targets from three GEO chips. **(C)** Construction of the GC-related targets network.

According to TCM theory, GC mostly manifests through syndromes of spleen-stomach deficiencies and cold-and-blood-stasis. Considering the effects of DSD on warming meridians and dispersing cold and nourishing blood, we thus set out to evaluate the therapeutic effect of DSD on GC. Three gene expression datasets (GSE33335, GSE54129, GSE79973) derived from human GC and adjacent normal tissue were obtained from the Gene Expression Omnibus (GEO) database. The overlapped 189 differential-expressed targets among these datasets were collected as the “GC-related targets” for further analysis (Figures 2A–C). Detailed information of these GC-related targets are listed in Supplementary Table S2.

### Construction of Protein-Protein Interaction Networks and Enrichment Analysis of Core Targets for Danggui Sini Decoction in Gastric Cancer

**FIGURE 3:**
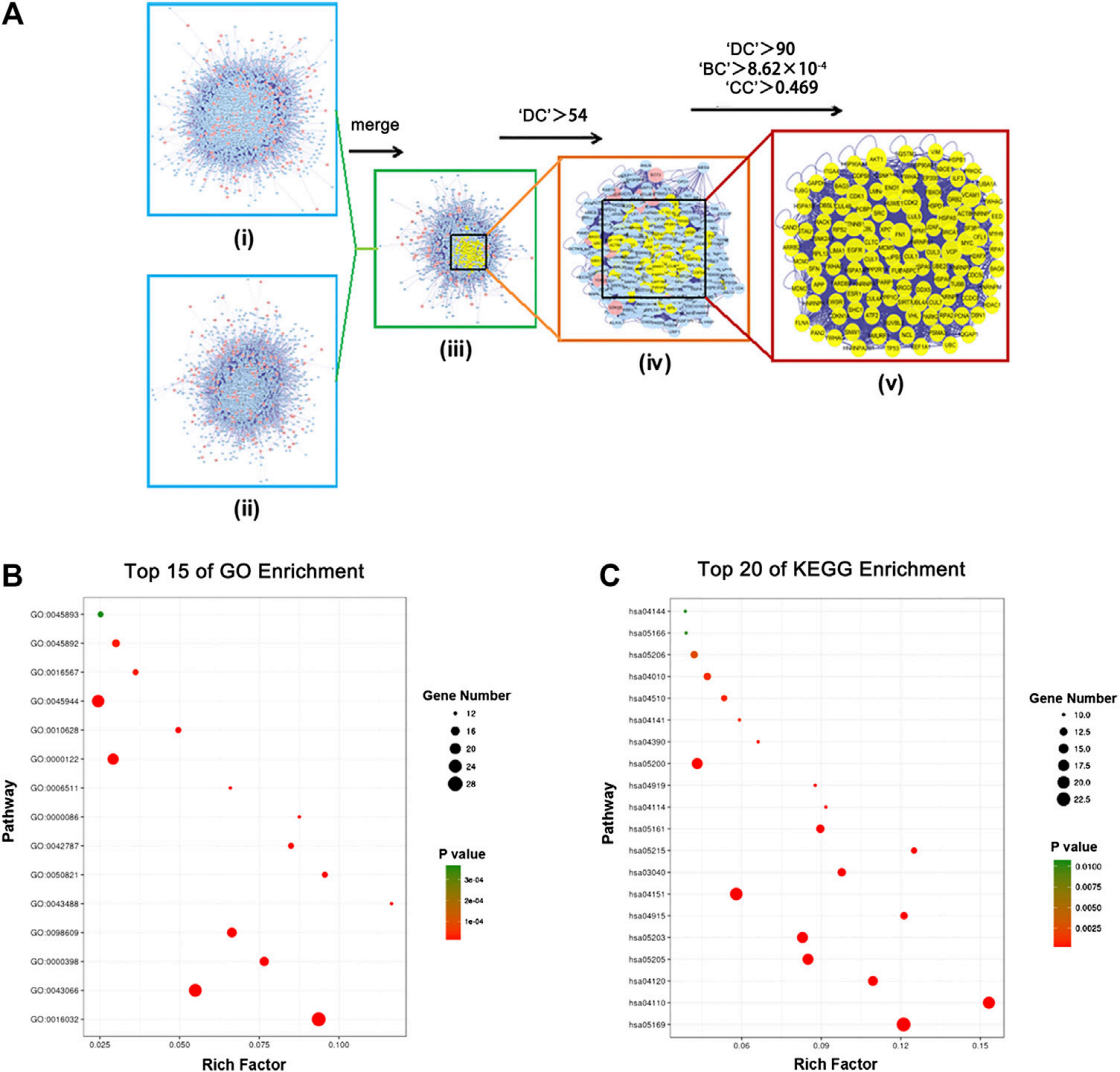
In silico identification and enrichment analysis of candidate core targets for DSD against GC. **(A)** i) The interactive PPI network of DSD putative drug targets was made of 4,961 nodes and 110,021 edges. ii) The interactive PPI network of GC-related protein targets was composed of 2,882 nodes and 50,906 edges. iii) The interactive PPI network of DSD putative drug targets and known GC- related targets made of 1,762 nodes and 39,450 edges was shown. iv) PPI network of significant targets extracted from iii, in which 460 nodes and 17,748 edges were included. v) PPI network of candidate core targets extracted from iv, in which 123 nodes and 2,808 edges were included. **(B)** Candidate core targets were enriched in the representative biological processes by using DAVID v6.8 (*p*-value < 0.05). **(C)** Candidate core targets were enriched in the representative signaling pathways by using DAVID v6.8 (*p*-value < 0.05).

To better understand the complex interactions among various targets, we constructed a putative drug-target PPI network for DSD, containing 4,961 nodes and 110,021 edges, as well as a GC-related target PPI network, containing 2,882 nodes and 50,906 edges, using the Bisogenet, a plugin for Cytoscape (3.2.1). Next, to investigate the mechanism-of-action of DSD against GC, we intersected these two networks and thereby obtained 1,762 nodes and 39,450 edges. Referring to a previous method, the topology parameter of each node in the overlapping network was calculated by using CytoNCA plugin, based on which a network of significant targets for DSD against GC, containing 460 nodes and 17,748 edges, was then constructed (Chen and Wang, 2012). To further uncover the key targets, we employed three topological parameters, namely DC, BC, and CC, to trim the number of nodes (core target) with higher parameter values, which eventually resulted in a collection of 123 core targets/nodes (Figure 3A; Supplementary Table S3).

We subsequently performed an enrichment analysis for these 123 core targets by dividing them into GO biological processes and KEGG signaling pathways. Specifically, the obtained biological processes were mainly related to apoptosis, G2/M transition of cell cycle, viral process, protein ubiquitination, and transcription, while the affected signaling pathways mainly included PI3K-Akt signaling pathway, MAPK signaling pathway, cell cycle, pathways in cancer, viral carcinogenesis, p53 signaling pathway, and ubiquitin mediated proteolysis (Figures 3B,C; Supplementary Figure S3; Tables 2, 3). These results suggested that DSD may inhibit the growth of GC cells by impairing the major signaling pathways involved in cell proliferation, cell cycle, and apoptosis.

**TABLE 2:** GO enrichment analysis of potential core targets for DSD against GC.

Term	Gene count	*p*-value
GO:0016032∼viral process	28	3.28E-22
GO:0043066∼negative regulation of apoptotic process	25	1.96E-14
GO:0045944∼positive regulation of transcription from RNA polymerase II promoter	24	5.29E-07
GO:0000122∼negative regulation of transcription from RNA polymerase II promoter	21	2.33E-07
GO:0098609∼cell-cell adhesion	18	1.17E-11
GO:0000398∼mRNA splicing, via spliceosome	17	6.05E-12
GO:0045892∼negative regulation of transcription, DNA-templated	15	1.58E-05
GO:0050821∼protein stabilization	13	2.97E-10
GO:0042787∼protein ubiquitination involved in ubiquitin-dependent protein catabolic process	13	1.16E-09
GO:0010628∼positive regulation of gene expression	13	4.59E-07
GO:0016567∼protein ubiquitination	13	1.19E-05
GO:0045893∼positive regulation of transcription, DNA-templated	13	3.71E-04
GO:0043488∼regulation of mRNA stability	12	2.14E-10
GO:0000086∼G2/M transition of mitotic cell cycle	12	4.62E-09
GO:0006511∼ubiquitin-dependent protein catabolic process	12	8.86E-08

**TABLE 3:** KEGG enrichment analysis of potential core targets for DSD against GC.

Term	Gene count	*p*-value
hsa05169:Epstein-Barr virus infection	23	9.86E-15
hsa04151:PI3K-Akt signaling pathway	20	2.63E-07
hsa04110:Cell cycle	19	6.59E-14
hsa05205:Proteoglycans in cancer	17	1.56E-08
hsa05203:Viral carcinogenesis	17	2.23E-08
hsa05200:Pathways in cancer	17	1.13E-04
hsa04120:Ubiquitin mediated proteolysis	15	5.87E-09
hsa03040:Spliceosome	13	3.12E-07
hsa05161:Hepatitis B	13	7.98E-07
hsa04915:Estrogen signaling pathway	12	1.19E-07
hsa04010:MAPK signaling pathway	12	9.07E-04
hsa05206:MicroRNAs in cancer	12	0.002222
hsa05215:Prostate cancer	11	3.74E-07
hsa04510:Focal adhesion	11	6.34E-04
hsa04114:Oocyte meiosis	10	2.12E-05
hsa04919:Thyroid hormone signaling pathway	10	3.04E-05
hsa04390:Hippo signaling pathway	10	2.69E-04
hsa04141:Protein processing in endoplasmic reticulum	10	6.16E-04
hsa05166:HTLV-I infection	10	0.010134
hsa04144:Endocytosis	10	0.010632

### Oral Administration of Danggui Sini Decoction Suppressed the Growth of Xenografted Gastric Cancer Cells on Mouse

**FIGURE 4:**
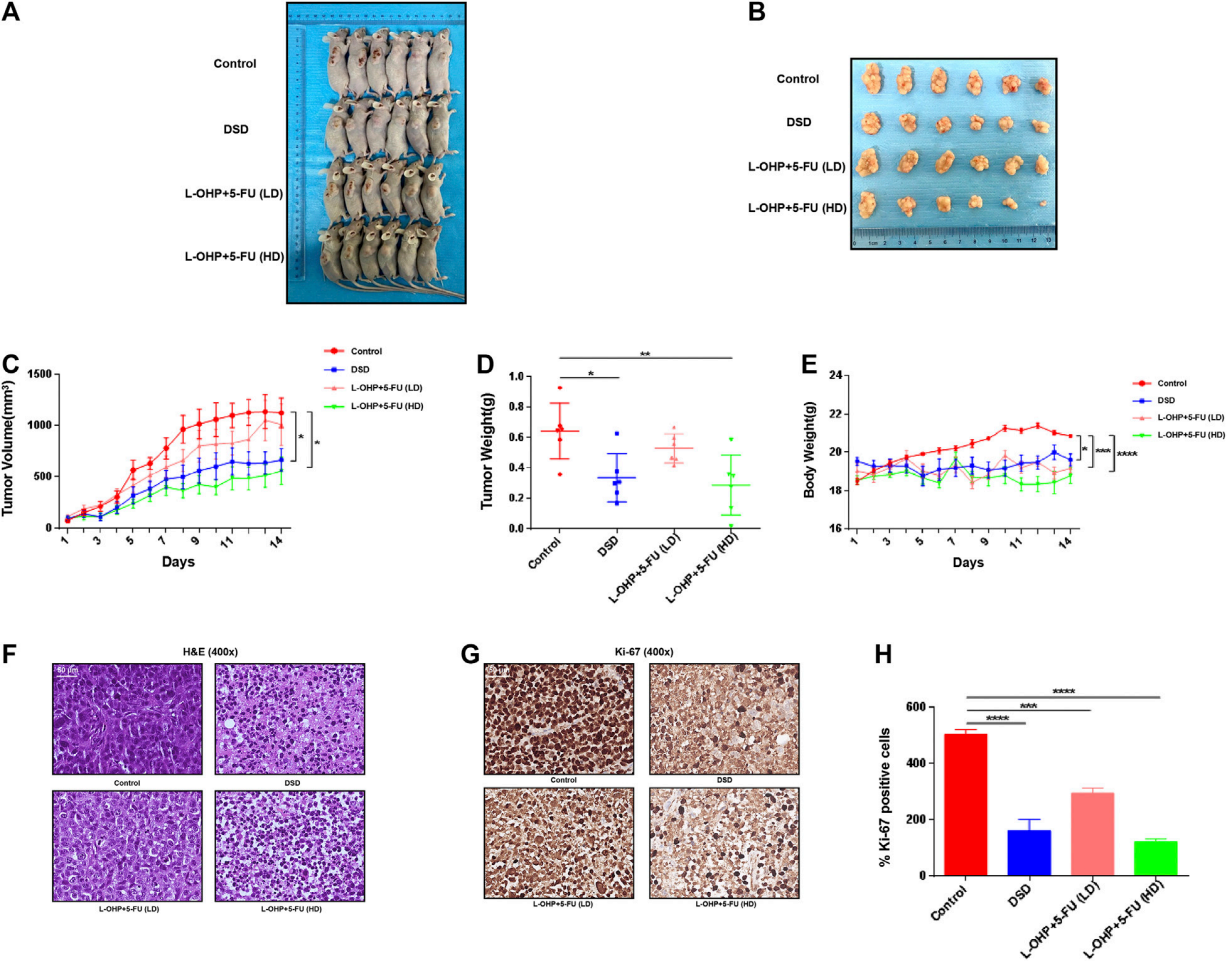
DSD suppressed development of the xenografted GC tumors on mice. **(A)** The tumor masses inoculated on BALB/c mice were carefully checked daily with the naked eye. The photos showed the mice bearing tumors on day 14. **(B)** The tumors were resected on day 14. **(C)** The tumor volumes were measured and calculated once daily for 14 consecutive days. **(D)** The resected tumors from different treatment groups were weighted on day 14. **(E)** The body weight of micewere measured and documented once daily. **(F–G)** H&E staining and immunohistochemical staining of Ki-67 protein in vital areas of tumor slicescollected from different groups of mice. **(H)** Statistical analysis of the positive ratio of Ki-67 staining. *p < 0.05, ***p < 0.001 and ****p < 0.0001 based on the Student’s *t*-test.

To experimentally verify the above *in silico* result, we next orally administrated DSD to the immunodeficient mice bearing xenografted GC cells. As shown in Figure 4, the tumor growths were greatly suppressed by oral DSD administration. At day 14, the tumor volumes of the DSD-treated group were more than 2-fold smaller than those of the control group (Figures 4A–C). Consistently, the tumor weights showed a striking difference between these two groups (*p* < 0.05) (Figure 4D). Furthermore, we compared the therapeutic efficacy of DSD on GC with the first-line chemotherapy regimen, L-OHP plus 5-FU. Since the tolerance of mice to platinum is relatively poor, we therefore further divided the mice into low dose L-OHP plus 5-FU and high dose L-OHP plus 5-FU groups, so as to give priority to the safety of the animals with due consideration to the effectiveness of the drug. The result showed that the xenografted GC tumors treated with DSD grew much slower than the low dose L-OHP plus 5-FU treated group, but faster than the high dose L-OHP plus 5-FU group (Figure 4C). In line with this, the average volume and weight of DSD-treated tumors were also between the low dose and high dose L-OHP plus 5-FU groups at day 14 (Figures 4A–D). Notably, the body weight of the mice was affected by either oral DSD or chemo-drugs administration as compared to that of the control group, but no significant difference was observed among the drug intervention groups (Figures 4A,E). Histologically, the DSD-or chemotherapy drug-treated tumor tissues revealed conspicuous necrotic cells, whereas tumors in the control group displayed vigorous cell growth (Figure 4F). The subsequent immunohistochemistry analysis showed that the number of Ki-67 positive cells was significantly decreased in DSD-or chemotherapy drug-treated tumors compared to those in controls, validating an anti-proliferative effect of DSD on these tumors (Figures 4G,H). Thus, these results provided convincing evidence showing that DSD possesses a direct anti-GC activity *in vivo*.

### Danggui Sini Decoction Inhibited the Cell Growth and Decreased the Viability and Motility of Gastric Cancer Cells

**FIGURE 5:**
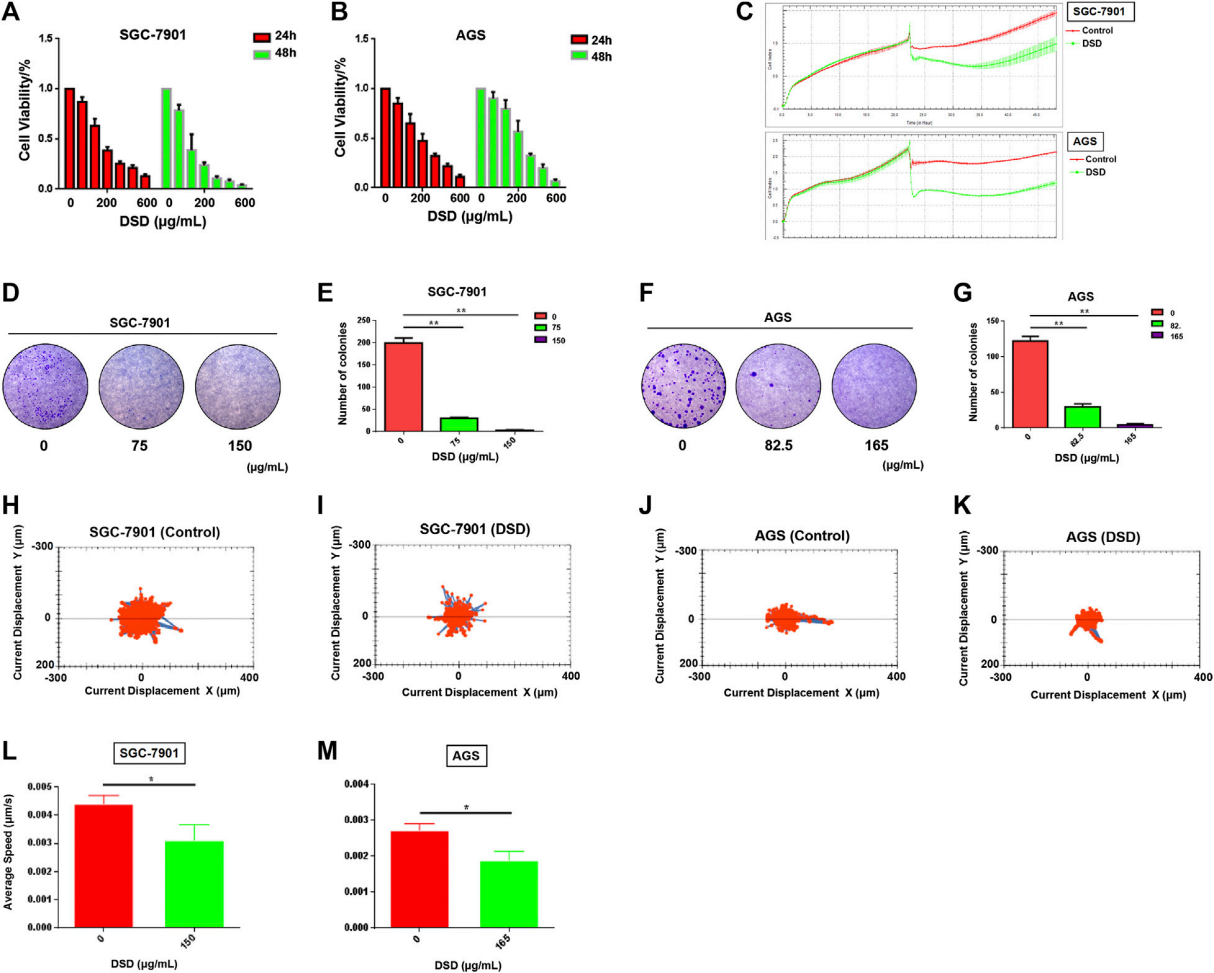
DSD inhibited GC cell proliferation and decreased its viability. **(A,B)** CCK-8 assay was performed to measure cell proliferation rate at 24 and 48 h after DSD treatment at different dosages (0, 50, 100, 200, 300, 400, 600 μg/ml). **(C)** xCELLigence RTCA was used to evaluate the drug toxicity at 24 h after DSD treatment at 150 μg/ml in SGC-7901 and 165 μg/ml in AGS. **(D–G)** Cell colony formation assay was performed to measure cell clonality after DSD treatment for 24 h. **p < 0.01 based on the Student *t*-test. **(H–M)** The Operetta CLS High Content Analysis System was used to evaluate the accumulated distance of cells at 24 h after DSD treatment at 150 μg/ml in SGC-7901 and 165 μg/ml in AGS.*p < 0.05 based on the Student *t*-test.

We next determined the pharmacological effect of DSD on GC cells. The results of CCK-8 assay showed that the growths of both SGC-7901 and AGS cells were significantly inhibited by DSD in a dose and time dependent manner (Figures 5A,B). After treating the cells for 24 h, IC_50_ analyses indicated that DSD exerted its 50% inhibitory effect on SGC-7901 cells and AGS cells at 153.50 ± 3.30 μg/ml and 167.30 ± 4.70 μg/ml, respectively. Based on the above IC_50_ values, we next evaluated the cytotoxicity of DSD to these GC cells using xCELLigence RTCA. The results from the dynamic monitor of the cytoactive cells for 24 h showed that the growths of GC cells were dramatically suppressed in the DSD treatment group compared to that in the control group (Figure 5C).

Furthermore, the viability of the GC cells were determined in the presence of DSD. The result of cell colony formation assay showed that the cell clonality of GC cells were decreased in a dose-dependent manner following DSD treatment for 24 h (Figures 5D–G). Meanwhile, the averages of accumulated distance of the migrating population in DSD treatment groups were shorter than control groups, indicating that the cell motility was inhibited by DSD (Figures 5H–M). Thus, these results demonstrated the direct suppressive effects of DSD on GC cell growth, viability, and motility *in vitro*.

### Danggui Sini Decoction Treatment Induced G2/M Arrest and Apoptosis in Gastric Cancer Cells

**FIGURE 6:**
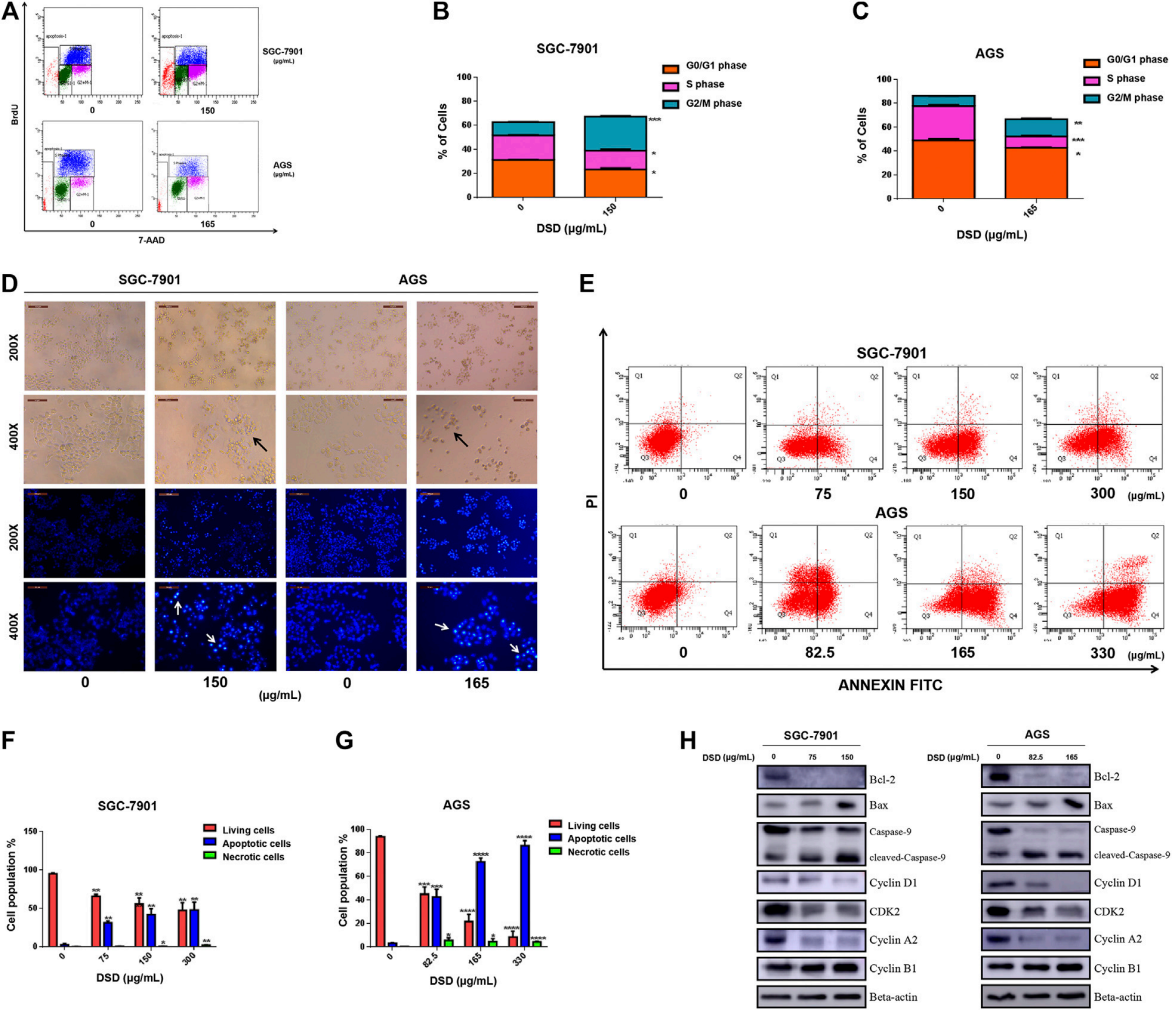
DSD treatment resulted in disturbance of cell cycle progression and apoptosis in GC cells. **(A)** Cell cycle analysis of GC cells following DSD treatment in SGC-7901 (0 and 150 μg/ml) and AGS (0 and 165 μg/ml) for 24 h by flow cytometry. **(B,C)** Statistical analysis of the proportions of the cells at different phases in SGC-7901 and AGS cells. **p < 0.01 based on the Student *t*-test. **(D)** Both cells’ morphology were observed in white light and fluorescence field using an inverted microscope. **(E)** Induction of apoptosis of SGC-7901 and AGS cells after DSD treatment. SGC-7901 cells were treated with DSD at different concentrations (0, 75, 150, and 300 μg/ml) for 24 h, and AGS cells were also treated with DSD at different concentrations (0, 82.5, 165, and 330 μg/ml) for 24 h, when apoptotic events was assessed by flow cytometry. **(F,G)** Statistical analysis of the percentages of the apoptotic cells in SGC-7901 and AGS cells. **p < 0.01 based on the Student *t*-test. **(H)** SGC-7901 (0, 75, and 150 μg/ml) and AGS (0, 82.5 and 165 μg/ml) cells were treated with DSD at different concentrations for 24 h. After proteins were extracted, the expression levels of Bcl-2, Bax, pro-caspase-9, cleaved-caspase-9, Cyclin D1, CDK 2, Cyclin A2 and Cyclin B1 were analyzed by Western Blot assay.

We next carried out a series of cellular functional assays to dissect the mechanism underlying the inhibitory effect of DSD on GC cell growth. Firstly, we performed BrdU-incorporated cell profiling assay to evaluate the effect of DSD on cell cycle. The results showed that DSD treatment significantly reduced the proliferation rate of GC cells by arresting them at G2/M phase (Figures 6A–C). Consistently, accumulation of the G2/M phase-specific marker Cyclin B1 and down-regulated Cyclin D1, CDK 2, and Cyclin A2 levels were observed in the DSD-treated GC cells (Figure 6H).

The morphology of GC cells was observed after DSD treatment for 24 h using an inverted microscope in either white light or fluorescence field. Compared with the control cells, the DSD-treated cells showed typical characteristics of cell apoptosis, such as shrinkage, roundness, and disappearance of stereopsis. Also, the DSD-treated cell nucleus showed dense Hoechst 33342 staining by fluorescence observation (Figure 6D). Moreover, flow cytometry analysis showed that the apoptotic population stained with Annexin V-FITC was significantly increased upon DSD treatment in a dose-dependent manner (Figures 6E–G). The subsequent western blot results confirmed that DSD promoted the accumulation of pro-apoptosis proteins Bax and cleaved Caspase-9 in a dose-dependent manner, while significantly down-regulating the level of anti-apoptosis protein Bcl-2 (Figure 6H). Together, the above results showed that DSD inhibited cell growth via induction of cell cycle arrest and apoptosis in GC cells, which further validated the anti-tumor effect of DSD on GC cells.

### Danggui Sini Decoction Inhibited Gastric Cancer Cells Through Akt/Erk/p53 Signaling Pathways

**FIGURE 7:**
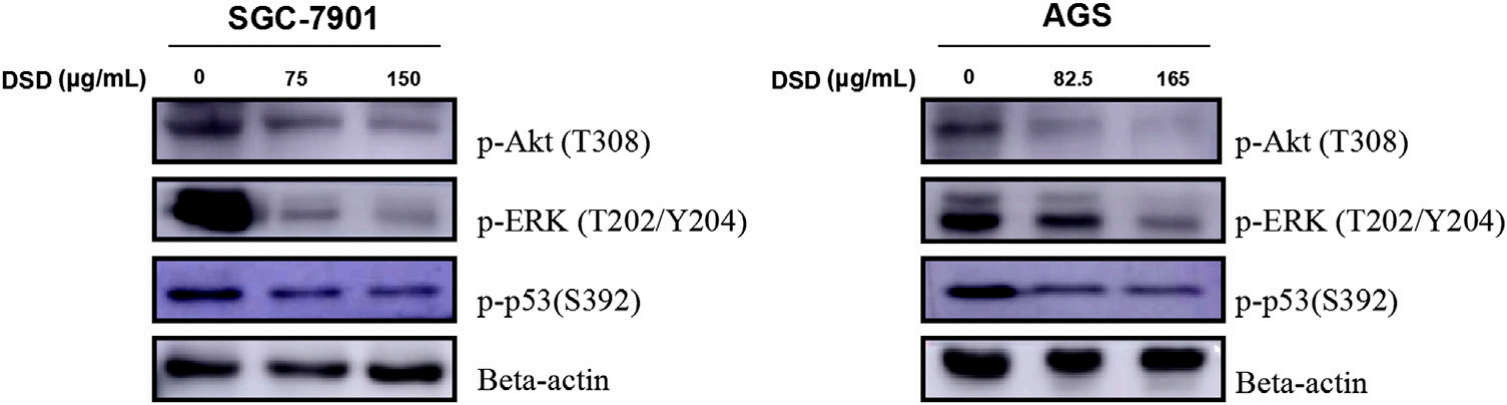
DSD treatment resulted in down-regulating the phosphorylation level of Akt, ERK, and p53 signaling pathways. SGC-7901 (0, 75, and 150 μg/ml) and AGS (0, 82.5, and 165 μg/ml) cells were treated with DSD at different concentrations for 24 h. After proteins were extracted, the expression levels of p-Akt (T308), p-ERK (T202/Y204) and p-p53 (S392) were analyzed by Western Blot assay.

To further investigate the action mechanism of DSD against GC cells, we set out to evaluate the activities of the signaling pathways that are closely related to cell proliferation and survival. Among those, PI3K-Akt, MAPK/Erk, and p53 signaling pathways were selected for further investigation based on the previous KEGG pathway enrichment analysis of the 123 core targets for DSD. Indeed, these pathways were significantly impeded upon DSD treatment, evidenced by dramatically down-regulated levels of phosphorylated forms of the key factors in these pathways in a dose-dependent manner, such as p-Akt (T308), p-ERK (T202/Y204), and p-p53 (S392) (Figure 7). Thus, these results suggested that the cell cycle arrest and apoptosis caused by DSD treatment resulted from simultaneous inhibition of Akt/Erk/p53 signaling pathways in GC cells, underscoring the “multi-active component, multi-drug target, and multi-function” characteristics of DSD.

### Molecular Docking Analyses of the Key Ingredients in Danggui Sini Decoction Suggested MCM2 as a Key Therapeutic Target Against GC

**FIGURE 8:**
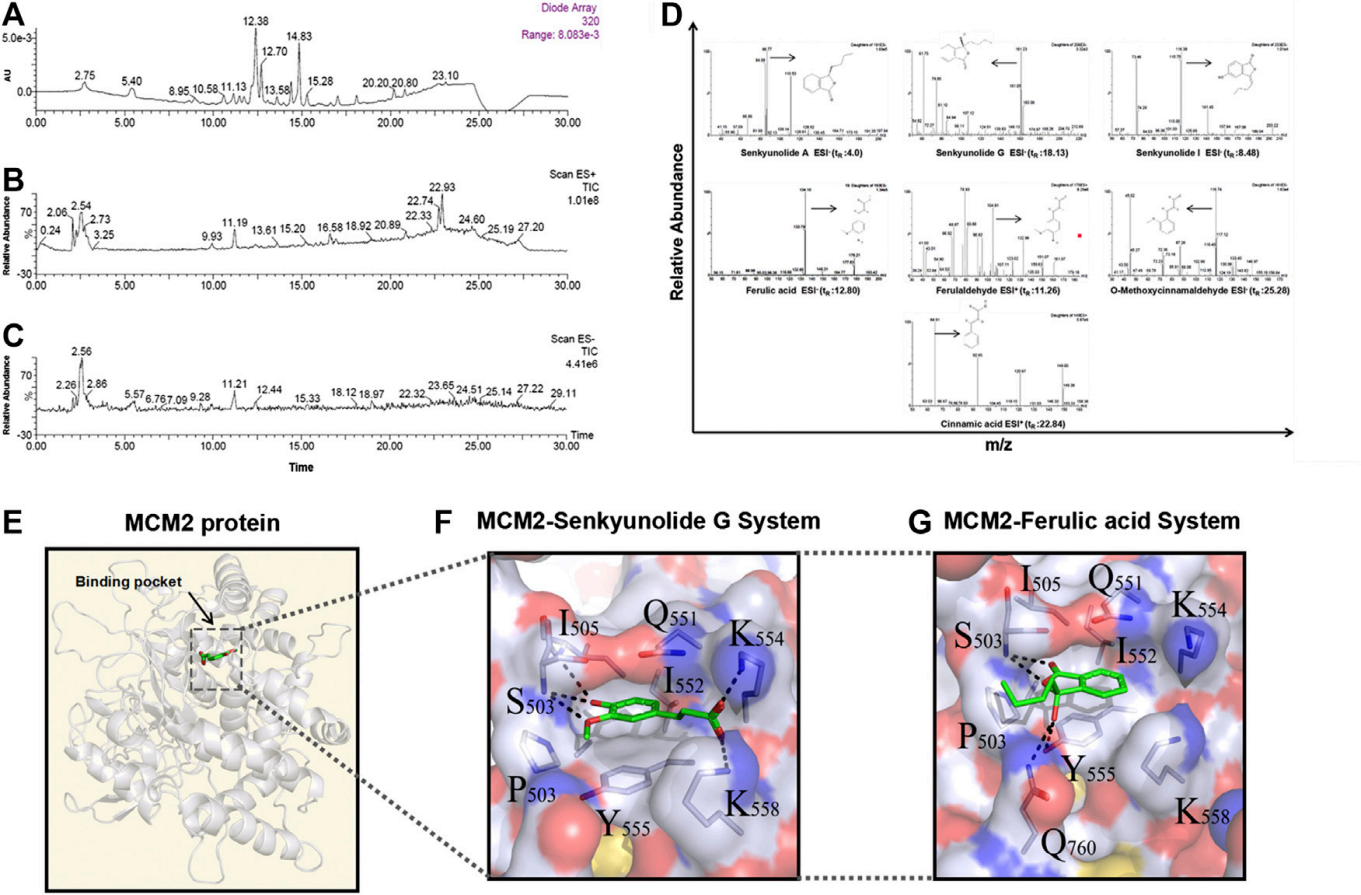
Seven main ingredients obtained from the two monarch drugs (DG and GZ) in DSD by using the LC-MS method and molecular docking analysis by AutoDock. **(A)** Ultraviolet absorption wavelength map. **(B)** Total Ion Chromatography (TICs) in positive ion modes. **(C)** Total Ion Chromatography (TICs) in negative ion modes. **(D)** Identification maps of the seven main ingredients by secondary mass spectrometry. Hydrophilic-hydrophobic interaction between **(E)** senkyunolide G, ferulic acid, and MCM2 protein binding pocket, and **(F–G)** senkyunolide G, ferulic acid and the surrounding amino acids in the MCM2 binding pocket.

Since the monarch herb in a CHM formula generally plays an essential role in delivery of its pharmacological effect, we next set out to identify the key ingredients in DG and GZ, two monarch herbs in DSD, using the UPLC-MS/MS method. Seven key ingredients from the crude extracts of DG and GZ were identified, including senkyunolide A (t_R_: 4.0 min), senkyunolide G (t_R_: 18.13 min), senkyunolide I (t_R_: 8.48 min), ferulic acid (t_R_: 17.09 min), ferulaldehyde (t_R_: 11.26 min), O-Methoxycinnamal (t_R_: 25.28 min), and cinnamic acid (t_R_: 22.84 min), which partially validated the key ingredients previously selected based on the ADME-related characteristics and literature searching (Figures 8A–D; Table 4).

**TABLE 4:** Identification of compounds in DSD by UPLC-MS/MS data.

No.	t_R_ (min)	Molecular formula	Selected ion	Theoretical	Experimental	MS/MS fragmentions	Compounds
1	4.0	C_12_H_16_O_2_	[M-H]^-^	192.25	191.35	110.53,86.77	Senkyunolide A
2	18.13	C_12_H_16_O_3_	[M-H]^-^	208.25	207	174.97,161.23,148.13	Senkyunolide G
3	8.48	C_12_H_12_O_3_	[M-H]^-^	204.23	203.22	189.04,116.38	Senkyunolide I
4	12.80	C_10_H_10_O_4_	[M-H]^-^	194.19	193.42	134.10	Ferulic acid
5	11.26	C_10_H_10_O_3_	[M+H]^+^	178.19	179.18	161.07,151.07,123.02,104.91	Ferulaldehyde
6	25.28	C_10_H_10_O_2_	[M-H]^-^	162.19	161	130.06,116.74,102.66	O-methoxycinnamaldehyde
7	22.84	C_9_H_8_O_2_	[M+H]^+^	148.16	149	120.97,92.95,64.91	Cinnamic acid

To further explore the action mechanism of DSD at the protein level, we performed molecular docking analysis for these key ingredients and the hub targets using Autodock 4.2. Firstly, the 123 core targets identified previously were ranked from high to low according to the values of their topological parameters “DC”, “BC”, and “CC”, respectively. The top 10 core targets from each ranking were selected and overlapped, which eventually resulted in six hub targets that appeared in all three top rankings, including EGFR, CUL3, APP, MCM2, CDK2, and FN1. Next, the docking efficiency between the above seven key ingredients and these six hub targets were predicted, among which the binding affinities of senkyunolide G and ferulic acid with a hub target MCM2 (energy: −7.44 and −6.63 kcal/mol, respectively) were greater than those between the other ingredients and targets (Table 5). The druggability analyses of senkyunolide G and ferulic acid revealed that the physical and chemical properties of both senkyunolide G and ferulic acid in the MCM2 protein binding pocket were stable, due to a good hydrophilic property of the pocket formed by hydroxyl/hydroxyl oxygen groups and ether bond. Meanwhile, the hydrophobic interaction could also be well formed between Benzene Ring in two ingredients and some other amino acids, such as Q551, I552, and Y555 in the pocket. Furthermore, the surrounding amino acids interacting with senkyunolide G and ferulic acid were mostly polar amino acids, such as Lys, Ser, Tyr, and Gln, which could form strong hydrogen bonds between these two ingredients and the residues around the binding pocket of MCM2 (Figures 8E–G). Thus, MCM2 is likely to be a key therapeutic target for senkyunolide G and ferulic acid against GC. However, further experimental verification is warranted.

**TABLE 5:** Results of molecular docking studies of seven compounds in the active sites of six proteins performed using Autodock 4.2.

Compound	EGFR	CUL3	APP	MCM2	CDK2	FN1
	(3W2S)	(4HXI)	(5BUO)	(3JA8)	(3MY5)	(3M7P)
Senkyunolide A	−5.72	−5.87	−5.33	−6.33	−5.43	−6.31
Senkyunolide G	−5.91	−6.31	−5.7	−6.63	−5.92	−5.1
Senkyunolide I	−6.44	−5.94	−6.3	−6.41	−6.22	−5.36
Ferulic acid	−6.5	−5.61	−6.46	−7.44	−7.01	−5.94
Ferulaldehyde	−5.82	−5.03	−5.2	−6.52	−5.98	−5.38
O-Methoxycinnamal	−5.01	−5.09	−5.37	−6.01	−5.68	−5.02
Cinnamic acid	−5.34	−5.23	−5.82	−5.82	−5.23	−4.97
						(kcal/mol)

## Discussion

TCM theory originated from ancient Chinese philosophy, and holds that all natural phenomena are composed of two complementary, interdependent, opposite, but exchangeable aspects, Yin and Yang (Tang et al., 2008). In view of TCM, Qi (energy) and blood circulate continuously through the so-called channels and collaterals in the body. Either imbalance of Yin–Yang or disturbed flow of Qi and blood may lead to disharmony of the organs, manifested as a variety of Zhengs (Su et al., 2014). Zheng, as a TCM term, is roughly equivalent to “syndrome” in western medicine. It can be defined by a series of TCM symptoms and signs, which are generally assessed by a unique set of methods, including looking, smelling, asking, and pulse feeling. Correspondingly, TCM practitioners make up a prescription based on the differentiation of Zheng rather than on a specific disease. However, the same Zheng can manifest in different diseases and vice versa (Chen and Wang, 2012). For this reason, although some classic CHM formulae have been used in folk medicine for hundreds of years, their clinical value may not have been fully appreciated. Thus, it is essential for the modernization of TCM to expand the clinical applications of classical CHM formulae by further exploring their clinical indications defined in modern medicine.

In fact, there is no such term as “gastric cancer” in classic TCM. Rather, according to the clinical symptoms, it has been expressed as “abdominal mass and gathering,” “dysphagia,” “epigastric pain,” or “stomach reflux.” For example, “the Synopsis of the Golden Chamber” describes: “vomiting breakfast in the evening, vomiting dinner the next morning, and indigestion of overnight food is called stomach reflux.” TCM theory believes that the development of gastric cancer is mainly due to congenital spleen-deficiency and other factors, such as diet and emotion. Of note, the word “spleen” in TCM is a term describing an entire group of physiological functions, rather than referring to a specific organ as in western medicine (Chen and Wang, 2012). In view of TCM, spleen is essential for the transformation of food into nutrients and Qi, which are then distributed to the other organs via blood and meridian. Therefore, spleen-deficiency would compromise the process of food digestion and nutrient absorption, causing cold coagulation in the meridian of stomach, which may lead to blood stasis, and eventually develop into “abdominal mass and gathering” over time. At present, with reference to the Zheng differentiation criteria in the Cancer Treatment Guidance by Chinese Society of Traditional Chinese Medicine, gastric cancer can be divided into six syndrome types, including spleen-stomach-deficiency cold syndrome, Qi stagnation and blood stasis syndrome, Qi-blood deficiency syndrome, liver Qi invading stomach syndrome, stomach heat injury Yin syndrome, and phlegm dampness coagulation syndrome. Among these, spleen-stomach-deficiency cold syndrome accounts for the majority of cases.

Like most of the classical TCM formulae, DSD is formulated in accordance with the TCM principle of monarch, minister, assistant, and envoy (Gao and Wu, 2008). Among the seven herbs contained in DSD, DG and GZ are the monarch herbs. According to the TCM theory, DG is sweet and mild in nature, with the function of nourishing and reconciling Qi and blood, while GZ is pungent and warm, with the function of warming meridian. Meanwhile, XX helps GZ to warm the meridian, and SY helps DG to nourish Qi and blood, both of which serve as the minister herbs. Moreover, TC has the function of dredging meridians, and DZ can replenish Qi and nourish blood, both of which are the assistant herbs. In addition, GC is used as the envoy herb to reconcile the properties of the other six herbs in the formula. Together, the overall effect of DSD is to warm meridian and dispel cold, while nourishing Qi and blood at the same time. Therefore, it is in line with the TCM theory that DSD can be used in the treatment of GC patients, such as those with spleen-stomach-deficiency cold syndrome.

In the present study, we first conducted a virtual study with the assistance of network pharmacology and bioinformatic analysis tools, which suggested a novel application of DSD on cancer treatment. We then selected GC for further investigation according to the Zheng theory of TCM. Due to the complexity of the interaction between the diverse ingredients of DSD and the disease network of GC, we started to explore the action mechanism of DSD against GC with a TCM network pharmacology approach along with the informatics methods like bioinformatics and chemoinformatics, which have been lately developed and applied on the ingredient and ingredient-target interaction prediction for CHM (Li et al., 2010a; Li et al., 2010b; Lagunin et al., 2014; Zhang et al., 2014; Pan et al., 2018b; Li et al., 2018; Zheng and Li, 2018). Thus, taking advantage of these computational methods, we identified 123 candidate core targets for DSD against GC. The enrichment analyses for these targets showed severe disruptions of a panel of essential biological processes and signaling pathways in GC cells upon DSD treatment, including apoptosis, cell cycle, PI3K/Akt signaling pathway, MAPK/Erk signaling pathway, and p53 signaling pathway, underscoring typical “multi-ingredient, multi-target, and multi-function” pharmacological characteristics of DSD. Indeed, the cell morphology observation and flow cytometry analysis of Annexin-V staining and BrdU incorporation assays showed a significant increased apoptotic events and G2/M arrest upon DSD treatment. Also, our western-blot results showed that the level of p-Akt (T308), p-ERK (T202/Y204), and p-p53 (S392) were all dramatically reduced upon DSD treatment in GC cells, validating the above signaling pathway enrichment result. Consistently, a previous study has shown that ferulic acid, an key ingredient of DG, could suppress the proliferation of osteosarcoma cells via inhibiting PI3K/Akt pathway (Wang et al., 2016). Paeoniflorin, an active ingredient from SY, has been reported to inhibit the proliferation of either pancreatic or endometrial cancer cells via MAPK/Erk signaling (Yang et al., 2016; Zhang et al., 2017). Furthermore, the extracts of SY or its active ingredient paeonol could down-regulate the expression level of oncogenic protein p53, thereby inhibiting the CRC cells’ proliferation (Lin et al., 2014).

Importantly, our *in vivo* experiment demonstrated that oral administration of DSD could suppress the growth of GC cells on immunodeficient mice. Notably, DSD appeared to have a higher suppressive efficacy on GC growth than low dose L-OHP (4 mg/kg) plus 5-FU did. However, given that the dose and dosing frequency of DSD used in this study were much higher than those of chemo-drugs, further evaluation of the exact therapeutic effect of DSD in the clinical practice setting is warranted. In view of this, results from this study not only shed light on the mechanism-of-action of DSD, but also propose the need for a clinical trial to assess the clinical performance of DSD on GC patients with spleen-stomach-deficiency cold syndrome.

To further reveal the underlying mechanisms of DSD against GC at a molecular level, we undertook a chemoinformatics approach toward the seven key active ingredients in the two monarch herbs of DSD that have been validated by UPLC-MS/MS. The top six candidate docking targets were selected from the core targets based on the values of their topological parameters. Among the total 42 molecular dockings, the top two binding affinities came from the interaction between senkyunolide G and MCM2 as well as ferulic acid and MCM2. Both senkyunolide G and ferulic acid bound to a common target with high binding affinities, suggesting a key role of MCM2 in delivering the pharmacological activity of DSD. As a member of the minichromosome maintenance (MCM) family, MCM2 is one of the subunits of the MCM2-7 complex that plays an essential role in DNA replication initiation and cell proliferation (Labib et al., 2000). MCM2 could be used as a prognostic marker for various malignancies, such as cervical carcinoma, oral squamous cell carcinoma, breast cancer, and gastric cancer (Gonzalez et al., 2003; Liu et al., 2013; Filho et al., 2014; Razavi et al., 2015). A recent study showed that MCM2 was implicated in lung cancer as an oncogene. Knockdown of MCM2 could dramatically inhibit cell growth via down-regulation of p53 signaling (Wu et al., 2018). Thus, it is rational to postulate that senkyunolide G and ferulic acid may convey the anti-proliferative effect of DSD on GC cells through binding with MCM2 to inhibit the p53 signaling. MCM6, another subunit of the MCM2-7 complex, was recently found to be up-regulated in hepatocellular carcinoma (HCC). Knockdown of MCM6 significantly decreased the proliferative ratio and invasive capability of HCC cells by impeding MEK/Erk signaling (Liu et al., 2018). Moreover, MCM7 participated in regulating cell growth and cell cycle progression in non-small-cell lung cancer through a MCM7/RACK1/Akt signaling complex (Fei et al., 2017). In this signaling complex, RACK1 functions as a scaffold protein to mediate the interaction between Akt and MCM7 to promote Akt-dependent MCM7 phosphorylation, which in turn facilitates MCM2-7 complex formation and subsequent DNA replication and cell proliferation. Together, results from these studies support a view that MCM2 may crosstalk with the PI3K/Akt signaling pathway or Erk signaling pathway via other subunits in MCM complex, through which it controls cell proliferation.

## Conflict of Interest

The authors declare that the research was conducted in the absence of any commercial or financial relationships that could be construed as a potential conflict of interest.

## Data Availability Statement

The original contributions presented in the study are included in the article/Supplementary Material, further inquiries can be directed to the corresponding authors.

## Ethics Statement

The animal study was reviewed and approved by The Institution Animal Care of Tianjin Medical University Cancer Institute and Hospital.

## Author Contributions

LL designed and supervised the study, summarized data from all contributors, and wrote the manuscript. BP engaged in study design, performed the network pharmacology and bioinformatics analysis, carried out the experimental validation, and drafted the manuscript. SF participated in study design and conducted the molecular docking analysis. YW contributed to the theoretical interpretation of our findings in the view of TCM. CW, JJ, and CH assisted with performing the experiments and bioinformatics analysis.

## Funding

This study was funded by the National Natural Science Foundation of China (no. 81572416), the National Key Technologies R&D Program of China (no. 2016YFC1303200), and the Tianjin Medical University Cancer Institute and Hospital Cancer Translational Medicine Seed Funds (no. 1701-1).

## Abbrevations

GC, gastric cancer; DSD, Danggui Sini Decoction; CHM, chinese herb medicine; TCM, traditional chinese medicine; OS, overall survival; CIPN, chemotherapy induced peripheral neuropathy; FBS, fetal bovine serum; CCK-8, cell counting kit-8; OB, oral bioavailability; DL, drug-likeness; PPI, protein-protein interaction; DC, degree centrality; BC, betweenness centrality; CC, closeness centrality; MDR, multi-drug resistant; LC-MS, liquid chromatography, mass spectrometric conditions; TICs, total ion chromatography.
