# Supplementary figures and images for: A Mechanism of Action Study on Danggui Sini Decoction to Discover Its Therapeutic Effect on Gastric Cancer
Source: Front Pharmacol. 2021 Jan 11;11:592903. doi: 10.3389/fphar.2020.592903 (PMC7830678; doi:10.3389/fphar.2020.592903)

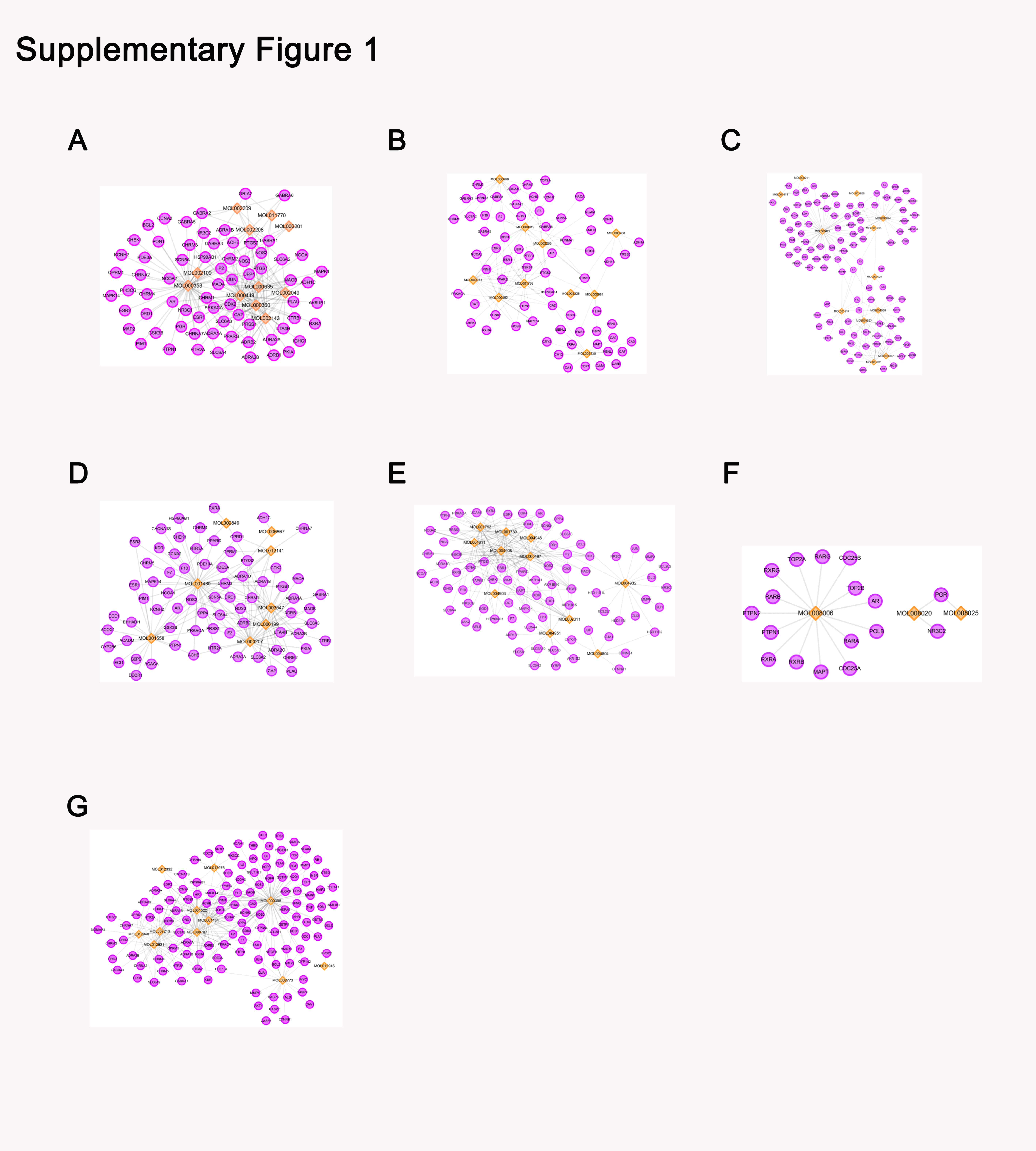

Supplement: Supplementary file 1 [file image1.tif]

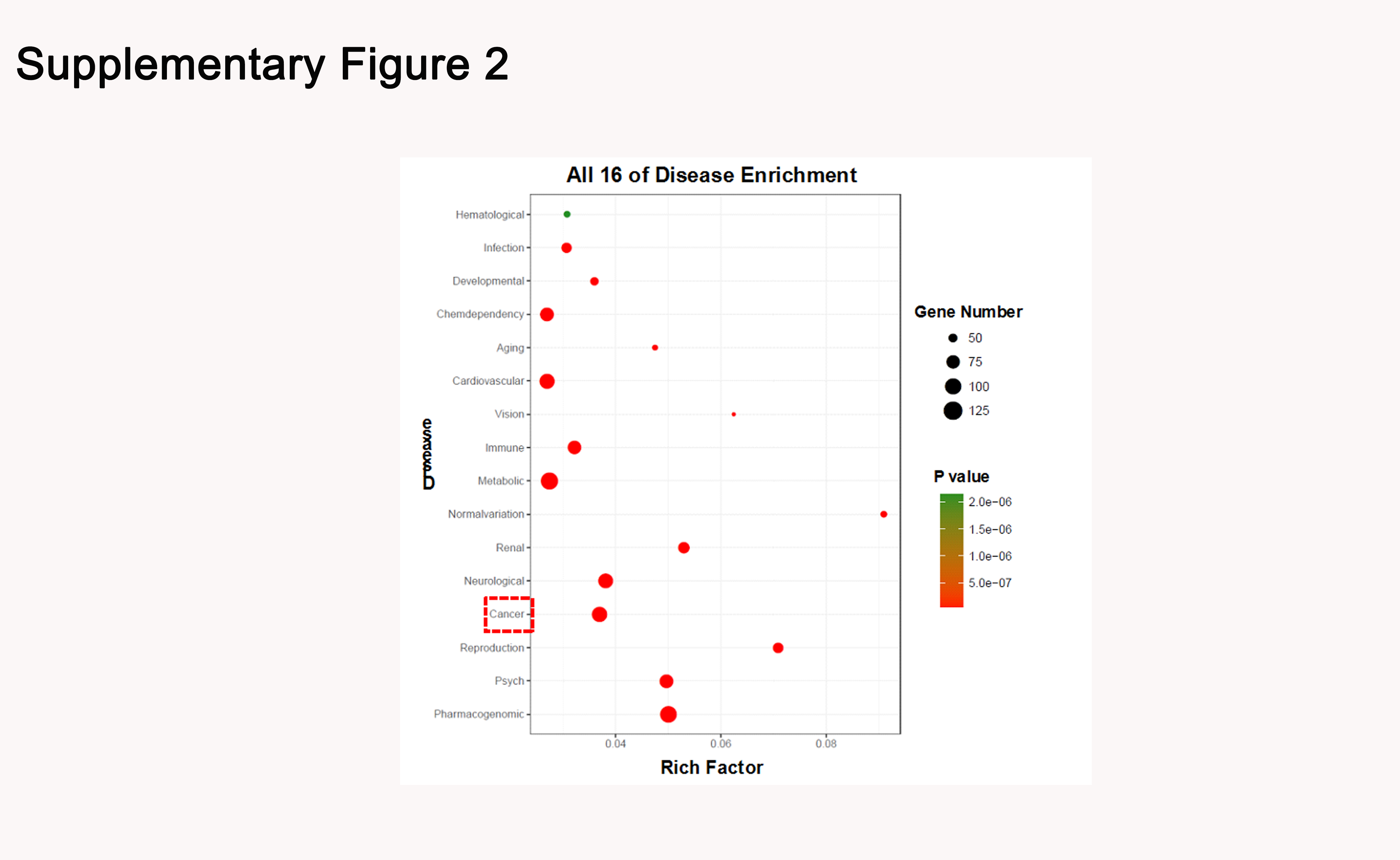

Supplement: Supplementary file 2 [file image2.tif]

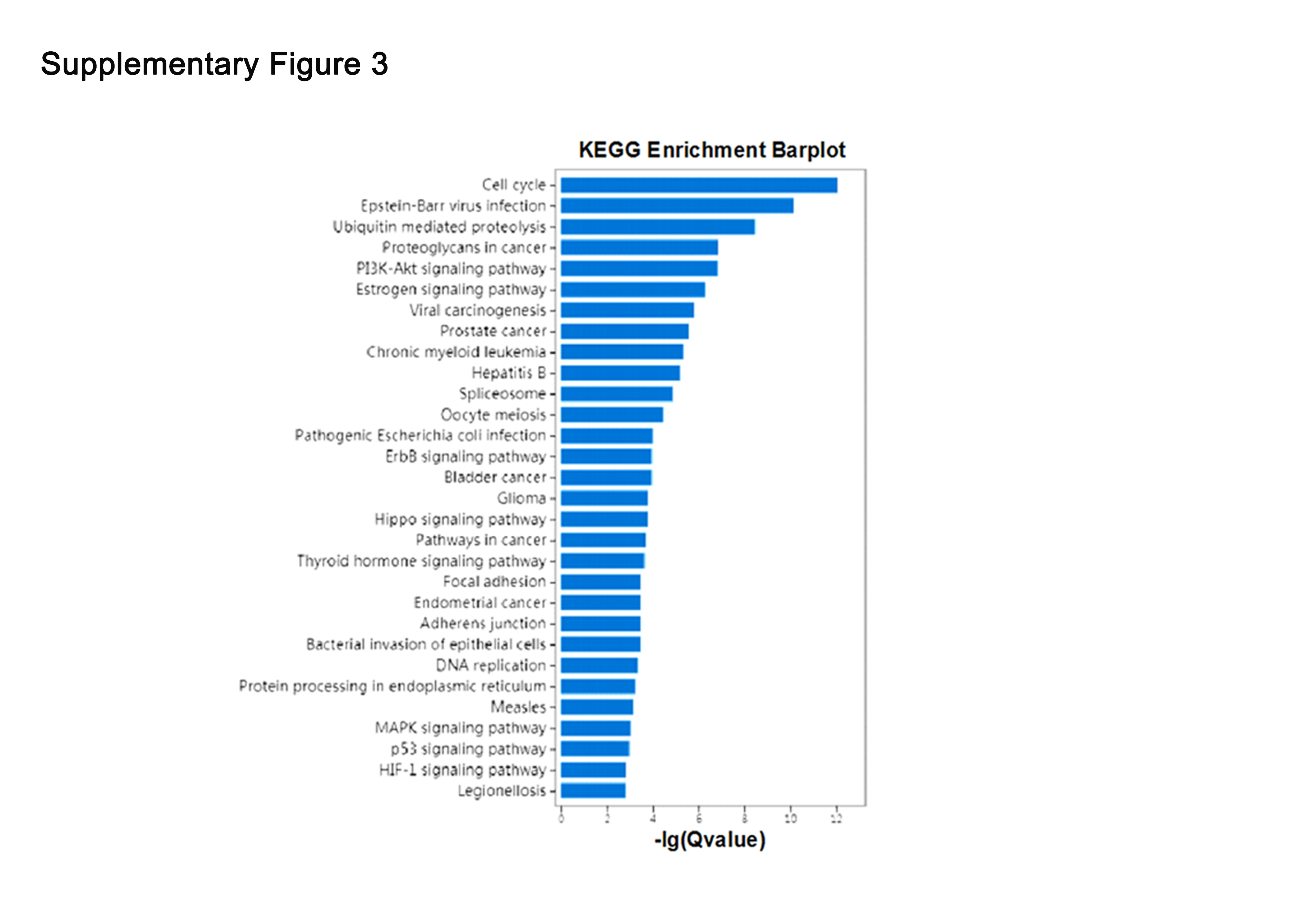

Supplement: Supplementary file 3 [file image3.tif]
